# Modified protocol for *in vivo* imaging of wild-type mouse retina with customized miniature spectral domain optical coherence tomography (SD-OCT) device

**DOI:** 10.1186/1480-9222-14-9

**Published:** 2012-10-11

**Authors:** Lee R Ferguson, Sankarathi Balaiya, Sandeep Grover, Kakarla V Chalam

**Affiliations:** 1 Department of Ophthalmology, University of Florida College of Medicine, 580 W 8th Street, Tower 2, 3rd floor, Jacksonville, FL 32209, USA

**Keywords:** Spectral domain optical coherence tomography, Customized, Retinal imaging, Mouse

## Abstract

This protocol outlines and evaluates a modified scanning procedure for a customized spectral domain optical coherence tomography (SD-OCT) imaging apparatus within the wild-type C57Bl/6 mouse posterior segment. This modified protocol allows for the capture of a 50 degree field of view spanning 3 mm by 3 mm perimeter with the optic disc as the central point. By utilizing this scanning protocol a more reliable measurement of retinal thickness can be achieved outside the fluctuating region of the optic disc. This protocol, when applied to this high resolution device, enables non-invasive *in vivo* histological imaging and biometric assessment of the various layers of the rodent posterior segment within a 20 – 30 min procedural time-frame. This protocol could establish a standardized method for evaluating morphological changes, with this commercial SDOCT device, when assessing longitudinal disease pathophysiology and treatment response in mouse models for future vision science research.

## Background

Mouse models represent valuable paradigms for studying retinal neurodegenerative and vascular disorders that are analogous in humans. By characterizing the natural course of pathological change evident in these models, information pertaining to morphology and function can be ascertained for individual diseases. Mouse models have been instrumental for understanding pathophysiology in a variety of retinal diseases including: retinopathy of prematurity [1], diabetic retinopathy [2-4], exudative age-related macular degeneration [5], retinal vascular occlusion and ischemia-reperfusion injury models [6] and inherited retinal diseases [7-11].

The gold standard approach for studying retinal disease changes in these animal models has been through the use of *ex vivo* histological preparations after sacrificing the animal. This methodology is fraught with limitations as it provides one time observation and includes tissue damage, toxicity, laborious technical procedures, as well as tangential assessment of disease pathophysiology. *In vivo* techniques such as fundoscopy, confocal scanning laser ophthalmoscopy (cSLO), angiography, and electroretinography (ERG) allow longitudinal observation of dynamic functional and morphological changes within disease models, but do not provide histological evidence.

SD-OCT provides rapid, high resolution, cross-sectional images almost resembling histology images and assist in the diagnosis of posterior segment pathology at various time points [12,13]. Because of this ability to acquire high-definition cross-sectional images of the retina, in humans, SD-OCT machines are being increasingly used in clinical practice. SD-OCT has been applied to investigate degenerative conditions not only in humans but also other animal species [14-17]. SD-OCT provides histological – grade sections of the rodent posterior segment non-invasively. However, acquisition of images in animals is cumbersome as machines are specifically designed for human use.

In this report, we describe a modified scanning protocol for a stereotactic rotational multidirectional animal containment apparatus, with SD-OCT imaging capabilities, for the acquisition of high resolution scans of the ocular posterior segment in mice. Our objective was to evaluate the implementation of a modified scanning protocol on a commercially available miniature SD-OCT device customized for rodent retinal imaging. Current use of this SD-OCT apparatus relies on scanning protocols that isolate retinal areas only pertaining to scanning region of approximately 1.5 mm by 1.5 mm [18,19]. Anatomically, this represents sections of greater thickness fluctuations as the retina begins to bottleneck with the emergence of the optic disc at the posterior pole. In order to measure a more uniform retinal region, measurements taken outside this central subfield (CSF) area would permit for a more accurate determination of retinal thickness. In our study we implemented a scanning protocol which delineated the CSF area in order to assess retinal thickness from more uniform regions of the posterior segment.

## Results and discussion

**Figure 1:**
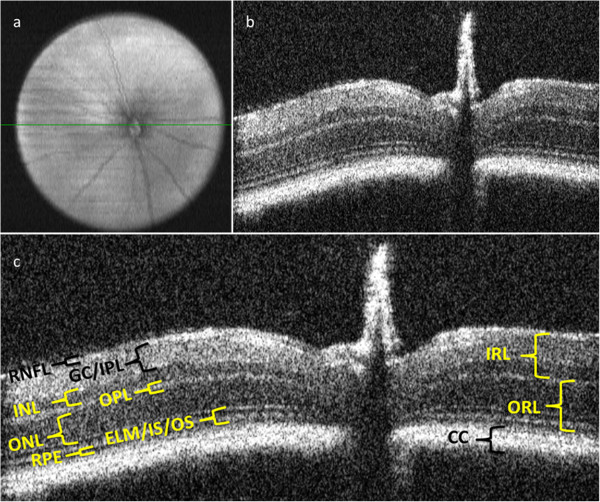
**C57Bl/6 retinal scans and posterior segmental layer thickness analysis. **En-face view of posterior segment with centralized optic nerve head (**a**). B-scan image of mouse retina from cross-sectional scan of en-face image (**b**). B-scan image with defined regions and thickness of posterior segment layers (**c**). CC: choriocapillaris, ORL: outer retinal layer, IRL: inner retinal layer, RPE: retinal pigment epithelium, ELM/IS/OS: external limiting membrane/inner segment of photoreceptors/outer segment of photoreceptors, ONL: outer nuclear layer, OPL: outer plexiform layer, INL: inner nuclear layer, IPL/GC: inner plexiform layer /ganglion cell, RNFL: retinal nerve fiber layer.

**Figure 2:**
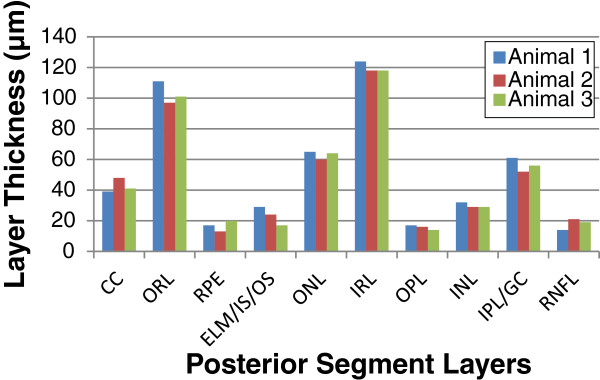
**Thickness measurements of C57Bl/6 posterior segment layers. **Thickness was calculated utilizing InVivoVue Clinic imaging software. Abbreviations represent the following: CC: choriocapillaris, ORL: outer retinal layer, RPE: retinal pigment epithelium, ELM/IS/OS: external limiting membrane/inner segment of photoreceptors/outer segment of photoreceptors, ONL: outer nuclear layer, IRL: inner retinal layer, OPL: outer plexiform layer, INL: inner nuclear layer, OPL: outer plexiform layer, IPL/GC: inner plexiform layer /ganglion cell, RNFL: retinal nerve fiber layer.

The Bioptigen animal imaging mount with rodent alignment stage (AIM-RAS) apparatus and miniature SD-OCT hand-held probe (HHP), allows for effective visualization of the posterior segment in wild-type C57Bl/6 mice. Figures 1A and 1B demonstrate en-face as well as B-scan images of a representative posterior segment scan from a C57Bl/6 wild-type mouse while utilizing the modified scanning protocol for the HHP. As depicted in Figure 1C, the retinal layers which were measured included the following: choriocapillaris (CC), outer retinal layer (ORL), inner retinal layer (IRL), retinal pigment epithelium (RPE), external limiting membrane/inner segment of photoreceptors/outer segment of photoreceptors (ELM/IS/OS), outer nuclear layer (ONL), outer plexiform layer (OPL), inner nuclear layer (INL), inner plexiform layer /ganglion cell (IPL/GC), and retinal nerve fiber layer (RNFL). Figure 2 graphically depicts the thickness measurements for three mice imaged with the AIM-RAS apparatus. Overall, the mean (range) value for the thickness measurements for the CC, ORL, and IRL were 42.67 μm (39 μm – 48 μm), 103 μm (97 μm – 111 μm), and 120 μm (118 μm – 124 μm) respectively. Measuring each layer of the retina individually, the mean (range) thickness values for the RPE was 16.67 μm (13 μm – 20 μm); ELM/IS/OS was 23.33 μm (17 μm – 29 μm); ONL was 63 μm (60 μm – 65 μm); OPL was 15.67 μm (14 μm – 17 μm), INL was 30 μm (29 μm – 32 μm), IPL/GC was 56.33 μm (52 μm – 61 μm), and RNFL was 18 μm (14 μm – 21 μm). The mean thickness magnitudes for the measured layers were not significantly different among the animals studied, F (2, 27) = 0.022 (p = 0.978).

The Bioptigen spectral domain ophthalmic imaging system (SDOIS) represents an alternative non-invasive *in vivo* imaging device which achieves high resolution histological – grade cross-sections along with en-face retinal imaging for the purposes of amassing volume intensity projection scans. The SDOIS possesses comparable axial resolution depths as observed with other high resolution OCT devices [14,15,18-21]. The use of this specific setup, along with the customized scanning protocol designed to capture full-field fundus images and cross-sectional views of the posterior segment, permits the acquisition of real-time SD-OCT scans of retinal morphology.

## Conclusion

In summary, this protocol details the animal and ophthalmic preparatory procedures as well as optimized technical method for acquiring retinal scans with the SDOIS. The imaging procedures as outlined in this protocol can be employed to assess normal mouse retinal anatomy along with rodent ocular disease model morphology when operating the SDOIS AIM-RAS setup.

## Methods

### Materials and supplies

Three adult C57Bl/6 mice (Jackson Laboratory, Bar Harbor, ME) were utilized for this study. All experiments were performed in accordance with the University of Florida institutional animal care and use committee guidelines and adhered to The Code of Ethics of the World Medical Association (Declaration of Helsinki) for animal experiments. The mice were housed and maintained in the University of Florida animal care services (ACS) facility and subjected to a 12-hour light/dark cycle with constant access to nourishments. All scanning and preparatory procedures were conducted in an ACS designated and approved procedural location.

### Animal preparation

Ketamine (Ketaject, 100 mg/mL, 80 mg/kg body weight) and Xylazine (Ana Sed, 100 mg/mL, 10 mg/kg body weight) anesthetics were purchased from Webster Veterinary (Devens, MA). Sterile NaCl 0.9% (wt/vol) was used in addition to Ketamine and Xylazine for creation of the anesthetic mixture. The Ketamine (16 mg/mL) and Xylazine (2 mg/mL) anesthesia stock reagents were diluted in sterile 0.9% NaCl solution. Mice were anesthetized by means of i.p. injection of Ketamine/Xylazine mixture. Afterwards, the animals were placed onto a 37°C thermostatic heating pad while in a sternally recumbent position. Once the mice were deemed fully anesthetized, one drop of dilating agent was applied for 2 – 3 minutes bilaterally. The dilating solution was wicked away and one drop of artificial tear was added to each eye to prevent corneal desiccation. Mydriasis was achieved with use of Tropicamide 1% (Akorn Inc.; Lake Forest, IL) while corneal hydration was maintained with Systane Ultra® lubricant eye drops (Alcon, Fort Worth, TX).

### Imaging and animal equipment

**Figure 3:**
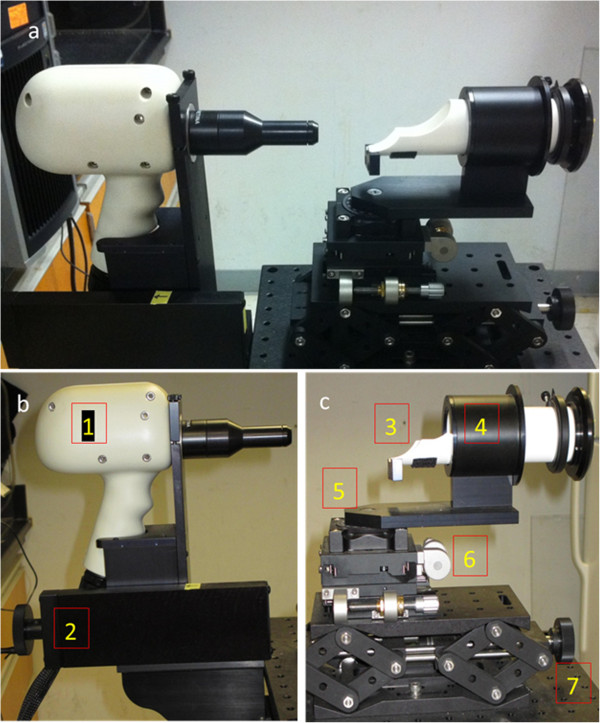
**Imaging setup. **Overview of the Bioptigen spectral domain ophthalmic imaging system (SDOIS) (**a**), Animal Imaging Mount (AIM) component (**b**), and Rodent Alignment System (RAS) component (**c**). AIM component includes miniature SD-OCT hand-held probe (1) and Z-translator (2). RAS component includes cassette (3), bushing (4), base (5), X-translator (6), and Y-translator (7).

**Figure 4:**
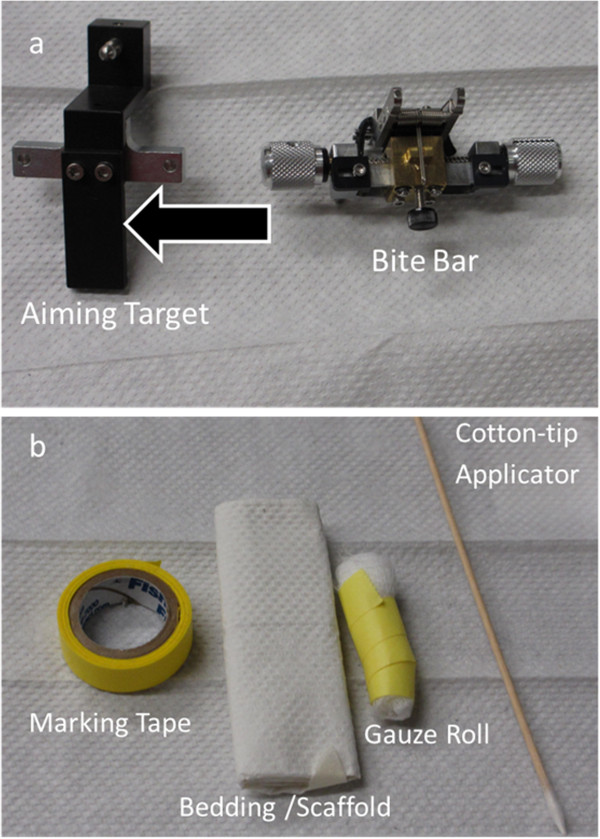
**Accessories for animal imaging mount with rodent alignment stage (AIM-RAS) setup. **Aiming target with tongue (arrow) and mouse bite bar (**a**). Marking tape, mouse bedding/scaffold, rolled up gauze for wedging mouse, and cotton-tip applicator for wicking eye drops (**b**).

The Bioptigen SDOIS (Bioptigen, Inc., Durham, NC) is a noninvasive imaging Class I, Type B, IPXO, continuous operation medical device. It delivers axial resolution capabilities of less than 6 microns and can process 20,000 A-scans/second. The SDOIS apparatus is comprised of a base system as well as an animal imaging mount and rodent alignment stage (AIM-RAS), which houses a SD-OCT hand held probe (HHP) (Figure 3a – c). The base system incorporates a host computer, 840 nm OCT engine with reference arm attachment, and the HHP. The HHP SD-OCT scanner was encased via the Animal Imaging Mount (AIM) (Bioptigen, Inc., Durham, NC), which allowed forward and backward adjustment of the HHP (Figure 3b). The InVivoVue Clinic software (Bioptigen, Inc., Durham, NC) enables the creation, display, loading and saving of OCT image files. The Rodent Alignment System (RAS) contained an X- (micrometer), Y- (scissor jack), and Z- translators along with stereotactic rotational cassette (for housing mice) within a bushing and platform base (Bioptigen, Inc., Durham, NC) (Figure 3c). The entire AIM-RAS device was attach to a slit-lamp base. The aiming tip, with cassette tongue, and aiming target were used for determining geometrical working distance for the optical lens bore to the mouse eye (Bioptigen, Inc., Durham, NC) (Figure 4a). In order to suppress breathing artifacts and provide fine tuning of horizontal and vertical positioning, a bite bar was employed (Bioptigen, Inc., Durham, NC) (Figure 4a). Cotton-tip applicators (Rush Dental & Medical Supply; Santa Monica, CA) were employed to wick eye drops (Figure 4b). In an attempt to wedge the mouse to a selective side of the animal cassette, as well as blanket the animal to maintain body warmth, surgical gauze was utilized (AllegroMedical) (Figure 4b). Surgical (3M™) and marking (Sigma) tape were applied so as to secure the mouse during rotational maneuvering and to identify the location of Z-translator when aligning the aiming tip to aiming target (Figure 4b).

### Mouse positioning

**Figure 5:**
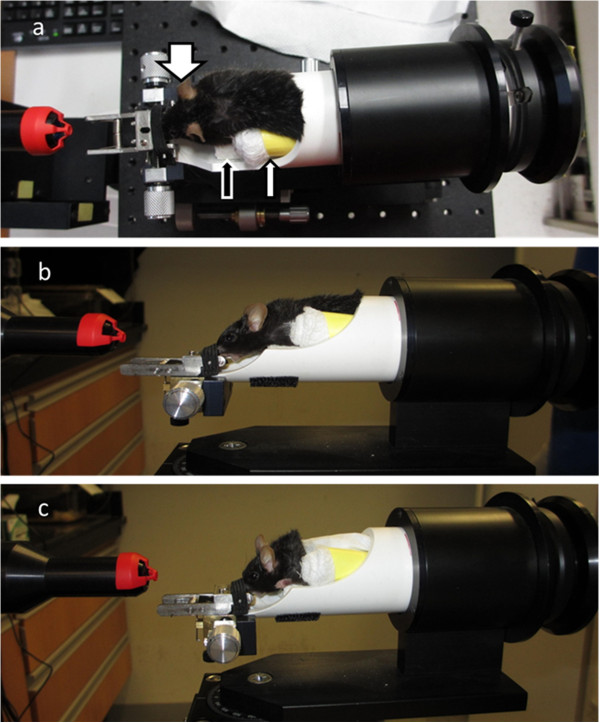
**Mouse insertion into cassette and attachment to bite bar. **Aerial view of mouse within cassette while attached to bite bar; large white arrow points to right eye where all scans were obtained from; small white arrow indicates gauze roll used for wedging animal to one side of the cassette; black arrow shows bedding/scaffold used to maintain straight and level head and body posture for the mouse (**a**). Lateral view of mouse within cassette (**b**). Lateral view of mouse blanketed with gauze to retain body heat (**c**).

The mice were positioned into the animal cassette according to body habitus. Depending on mouse body size, smaller mice were raised within the cassette by fashioning rectangular bedding made from gauze or folded paper towel. This assured that the mouse was positioned within a straight line and that the neck was level so that the head was looking straight forward. Also, depending on which eye was selected for scanning, a cylindrical gauze roll was created in order to bias the mouse body, and therefore eye, to one side of the cassette. Thus, when scanning the right eye, the animal was biased toward the left side of the cassette (Figure 5). Lubricant drops were evenly spread across the cornea during rewetting in order to eliminate optical aberrancies and ensure adequate focus during scanning of the retina. The mouse was secured within the cassette via comfortably strapping the animal with surgical tape. The HHP bore, with aiming target attached, was brought in close proximity to eye by adjusting the Z-translator on the AIM apparatus or by shifting the cassette within the bushing.

### SD-OCT imaging of ONH and retina

After the HHP lens was situated close to the right eye of the animal, the InVivoVue Clinic application was activated and the scanning began- following setup of subject profile for image acquisition. We selected the rectangular scanning protocol consisting of a 3 mm by 3 mm perimeter with 1000 A-scans per B-scan with a total B-scan amount of 100. This is a modification of the recommended parameters of 1.4 mm by 1.4 mm set by the company for performing rectangular scans.

In order to bring the ONH into view and focus we made fine tune adjustments to the animal cassette. At this point the eye was docked into the aiming target, and the ONH was centered within the viewing pane, of the InVivoVue Clinic application, for the B-scan and the en face images of the retina. We fine-tuned B-scan focusing by adjusting the Z-translator on the AIM, which moved the HHP towards or away from the eye, and also by dialing the reference arm on the base system in order to obtain bright crisp B-scan images. The CSF was defined as the central 1 mm area containing the ONH. This region was demarcated by using the InvVivoVue Clinic measurement calipers to establish the 1 mm diameter area from the center of the ONH. Thickness measurements were performed with the caliper instruments outside the CSF area.

### Statistical analysis

Statistical analyses included the mean and range for each of the posterior segment layers in the three animals examined. A one-way analysis of variance was performed to contrast layer thickness between the three animals. Statistical significance was achieved if the two tailed p-value was less than the alpha level of 0.05.

## Competing interests

The authors declare that they have no competing financial interests.

## Authors’ contributions

L.R.F. and S.B. prepped the animals, performed scans, and optimized the procedure. L.R.F. wrote the manuscript, collected the data, presented and prepared the figures. S.G. and K.V.C. assisted in structuring the content of the manuscript and revisions. K.V.C. supervised the work. All authors read and approved the final manuscript.
